# Microbial Colonization, Biofilm Formation, and Malodour of Washing Machine Surfaces and Fabrics and the Evolution of Detergents in Response to Consumer Demands and Environmental Concerns

**DOI:** 10.3390/antibiotics13121227

**Published:** 2024-12-18

**Authors:** Patricia Osta-Ustarroz, Allister J. Theobald, Kathryn A. Whitehead

**Affiliations:** 1 Lubrizol Life Science, Vanguard House, Keckwick Lane, Daresbury, Cheshire WA4 4AB, UK; patricia.osta-ustarroz@lubrizol.com; 2 Microbiology at Interfaces, Manchester Metropolitan University, Chester Street, Manchester M1 5GD, UK

**Keywords:** washing machines, detergents, laundering, fabrics, textiles, bacteria, sustainability, biofilms, consumers, malodour

## Abstract

Bacterial attachment and biofilm formation are associated with the contamination and fouling at several locations in a washing machine, which is a particularly complex environment made from a range of metal, polymer, and rubber components. Microorganisms also adhere to different types of clothing fibres during the laundering process as well as a range of sweat, skin particles, and other components. This can result in fouling of both washing machine surfaces and clothes and the production of malodours. This review gives an introduction into washing machine use and surfaces and discusses how biofilm production confers survival properties to the microorganisms. Microbial growth on washing machines and textiles is also discussed, as is their potential to produce volatiles. Changes in consumer attitudes with an emphasis on laundering and an overview regarding changes that have occurred in laundry habits are reviewed. Since it has been suggested that such changes have increased the risk of microorganisms surviving the laundering process, an understanding of the interactions of the microorganisms with the surface components alongside the production of sustainable detergents to meet consumer demands are needed to enhance the efficacy of new antimicrobial cleaning agents in these complex and dynamic environments.

## 1. Introduction

Laundering plays an important role in the control of both pathogenic and odour-causing microorganisms [1], and the removal of pathogens from laundry is largely dependent on washing and drying practices [2]. Sustainability trends have influenced users to wash at lower temperatures, with reduced water and energy consumption. In addition, there has been an increased use of bleach-free liquid detergents [3]. However, from a hygienic point of view, these adjustments can negatively affect laundry hygiene by facilitating the survival of microorganisms inside the washing machine and on washed laundry [4]. This review gives an introduction to washing machine use and the diversity of surfaces found in washing machines and discusses how biofilm confers survival properties to the microorganisms and leads to their production of volatiles. Changes in consumer attitudes, sustainably produced chemicals, and consumer demands with respect to laundering habits are further discussed.

## 2. Washing Machine Use and Surfaces

Most industrialized countries experienced a significant increase in the ownership of a washing machine during the 20th century. Countries such as China and India have exhibited a steady increase in the percentage of washing machine sales from 2004 (10–14%) to 2018 (40–45%) [5]. At the beginning of the 20th century, only about eight percent of USA families possessed a washing machine [6]. The latest market data show that the UK alone consumes over 2.6 million washing machines annually, and if the numbers extend to Europe, this can be predicted to be as many as 21 million washing machines per year [7,8]. With more people buying washing machines, it is expected that over 30 percent of the entire world’s population will own washing machines by the year 2025 [9].

### The Diversity of Surfaces Found in Washing Machines

**Figure 1:**
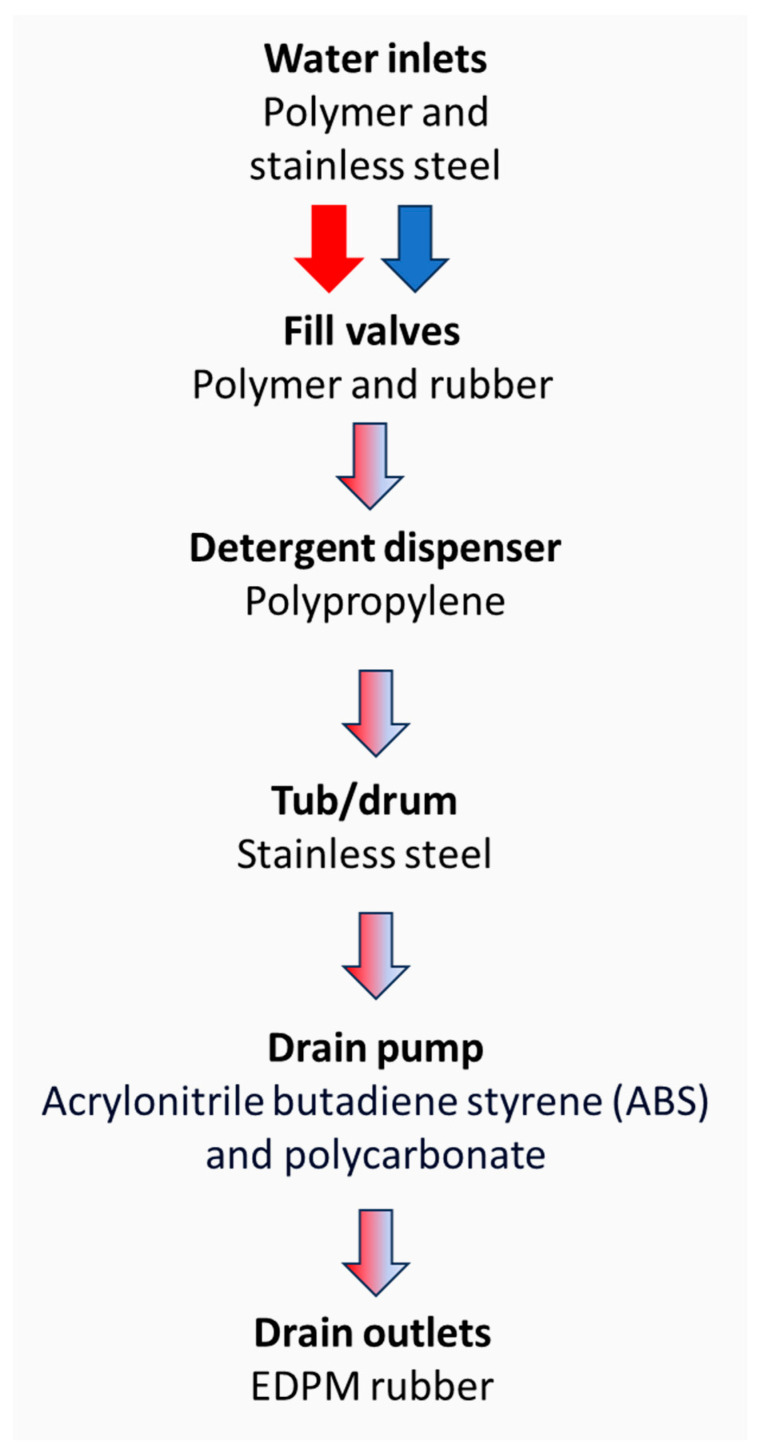
Movement of water through the chemically different components of a washing machine.

The main chemical components of a washing machine’s construction are stainless steel, synthetic rubber, and polypropylene (PP) (Figure 1). The inner drum of a washing machine, which is the element which moves around and is perforated with holes to allow the water in and out, may be manufactured from porcelain or plastic, but stainless steel is the most common material used due to its durability [10]. The ferritic stainless steel group (series 400), specifically stainless steel 430 (SS430), is a low-carbon, chromium ferritic stainless steel that has good formability and corrosion resistance, which makes it suitable for use in applications such as catering equipment, kitchen sinks, or washing machines [11]. The features of this stainless steel make it a beneficial option for use in washing machines since mild chemicals such as detergents and cleaning agents do not result in corrosion [12]. The washing machine seal around the door is generally made of a synthetic rubber, and prevents water from leaking out of the appliance. It is usually made from ethylene propylene diene monomer rubber (EPDM) [13]. This washing machine component presents specific characteristics such as a high tensile strength, elongation properties, and elevated rubber hardness. This makes it suitable for dealing with the working conditions of the drum washing machine, which results in vibrations, stretching, and compression of the door seal. Finally, polypropylene is the most common type of plastic used in the drain hoses and pipes of washing machines, although polyvinyl chloride (PVC), acrylonitrile butadiene styrene (ABS), and polycarbonate (PC) are also used. The pipework throughout the washing machine can be composed of a number of different shapes, angles, and diameters, which result in a range of flow types, rates, and environmental conditions.

**Figure 2:**
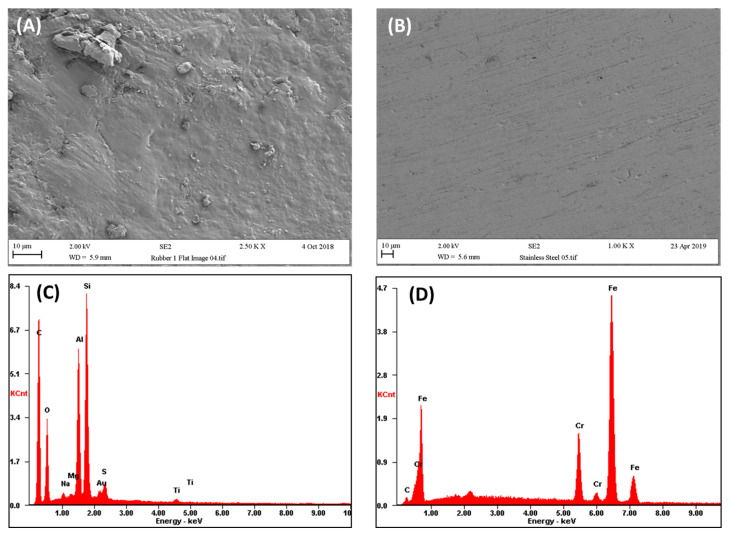
Different properties of some of the surfaces that can become biofouled during the laundering process: (**A**) topography of rubber seal, (**B**) topography of stainless steel, (**C**) elemental analysis of rubber seal, and (**D**) elemental analysis of stainless steel.

The main surfaces used in the washing machine have a range of roughness, topographies, and features which are dependent on their finish or method of production. The surfaces are also chemically different, which in turn can also influence their hydrophobicity and roughness. Polypropylene is a methylated form of polyethylene and is more hydrophobic (96.3°) than stainless steel (70–75°), and both are more wettable than EDPM (101.2°) [14]. Gattlen et al. [15] found as many as 15 key points inside a washing machine where biofilm could form and be harboured. Those locations included the detergent drawer, crossbar, pump, filter, rubber ring of the door, the drum, and the hoses. Although a number of suggestions have been made regarding recent innovation to reduce biofouling, such as antimicrobial coatings or nanocomposites, the availability of information pertaining to their use in washing machine testing is limited. The chemical matrix of the washing machine components is important since work has shown that when samples were tested from different sites in a washing machine, the bacterial community was found to be significantly site-dependent, with the highest diversity found inside the detergent drawer, followed by the sump, textile fibres isolated from the washing solution, and the door seal [16] (Figure 2). 

## 3. Biofilm Development

Microorganisms are present everywhere in nature, and generally their interactions with humans or animals are harmless [17]; however, some bacterial species are opportunistic and/or pathogenic. Washing machines are well-known to be reservoirs of microorganisms [18,19], and in the domestic environment, they can grow on a variety of materials, especially metal and rubber [20]. The microorganisms found in biofilms differ profoundly in a number of ways from their planktonic counterparts [21]. The transition from floating or planktonic cells to biofilm growth occurs in response to environmental factors or local conditions that trigger the transition of bacteria onto a surface and results in new emerging properties of the biofilm community [22].

**Figure 3:**
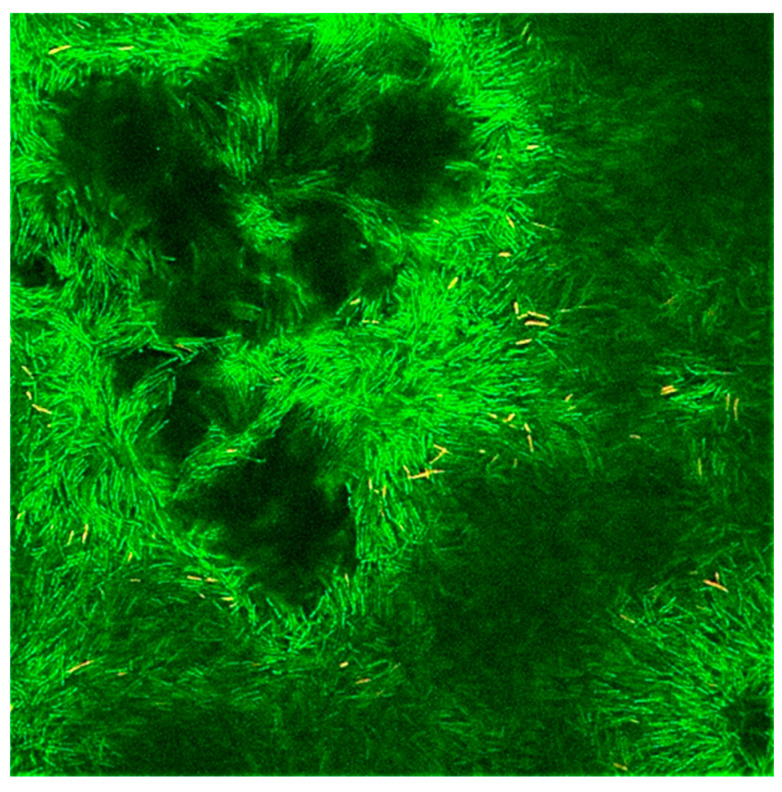
Biofilm formed by *Pseudomonas aeruginosa* demonstrating the heterogeneity of the biofilm formation and presence of water channels.

Biofilms can be defined as multicellular structured communities of bacterial cells held together and enclosed by a self-produced extracellular polymeric matrix (EPS) [23], forming aggregates of microorganisms that are adherent to each other and to a surface. Biofilms can be formed by a single species or by many species (as typically found in nature), which may include bacteria, algae, yeast, and fungi. Biofilms may form on a wide variety of surfaces, including fabrics; industrial or potable water system piping; natural aquatic systems [24], or within household devices such as dishwashers [25], showerheads [26], and washing machines [18]. The EPS that surrounds microorganisms within the biofilm is composed mainly of polysaccharides, lipids, proteins, and nucleic acids [27]. This matrix provides structural support for the biofilm and enables the microbial community to develop complicated three-dimensional entities. Within the biofilm, a network of open water channels can be found in which microbes interact among themselves, whereby they can permit an exchange of nutrients and/or metabolites [17] (Figure 3). This includes the use of molecules for cell-to-cell communication, commonly known as quorum sensing [28].

Biofilm formation can be considered a strategy by which microorganisms enhance their survival, and this enables them to adapt to the evolving environment, particularly in adverse and harsh conditions. Thus, their formation confers bacteria with many possible advantages compared to a planktonic existence, particularly in terms of microbial survival, growth, and dispersal [29]. The biofilm matrix further provides microorganisms protection from external forces and chemical factors such as biocides and disinfectants. Additionally, biofilms can increase cellular resistance to antibiotics [23]. Since the development of a biofilm results in unique cellular phenotypes that are different from planktonic cells, this may result in a genetic diversification of bacteria within the biofilm [30] or the development of new metabolic networks (changes in bacterial gene expression), which can cause spatial and temporal reorganization of the bacterial components in the biofilm [31]. Therefore, the biofilm constitutes a well-coordinated, functional community that can be much more efficient than planktonic microorganisms. There are various viewpoints on the point at which the retained cells start to become a biofilm. However, generally, the steps involved in biofilm formation begin with an attachment phase, whereby the bacteria are reversibly attached to the surface, followed by more firm adhesion of the bacteria onto the surface, then by bacterial retention, which constitutes irreversible binding of the bacteria. The cells then begin to proliferate, and as the biofilm phenotype matures, communities (aggregations of cells into microcolonies) are formed (Table 1).

It is known that differences in pH, temperature, and water composition affect biofilm formation, but information that specifically relates to how biofilm formation occurs in washing machines is unclear. Temperature is important to control pathogens, and temperatures exceeding 40 °C to 60 °C are required to inactivate several pathogenic species [2]. If temperatures below 20 °C are used, then other washing agent ingredients such as enzymes and chlorine and activated oxygen bleach are needed to reduce certain enteric viruses and bacteria [2]. However, at ambient temperatures, it has been demonstrated that a persistent microbiome may remain on laundry items and on the water-contact surfaces of the washing machine itself following regular washing cycles and continued use [3]. It has been suggested that this could result in continuous repopulation of the washing machine microbiome from items such as clothing being laundered and that there may be a potential for microorganisms from the machine microbiome to contaminate the clothes being washed [4]. Jacksch et al. [16] demonstrated that within a washing machine, there was a higher diversity of species with an increased number of wash cycles compared to a lower number of wash cycles at high temperature; this was also seen where there was an increased number of washing cycles per month. However, factors such as the age of the machine or regular cleaning measures did not have an effect on bacterial diversity [16]. Interestingly, Hamada and Abe [32] found that some fungal species could use anionic and non-ionic surfactants as nutrients, and other fungal species have been shown to be able to grow on media containing surfactants [33].

### 3.1. Biofilms and Surface Interactions

**Figure 4:**
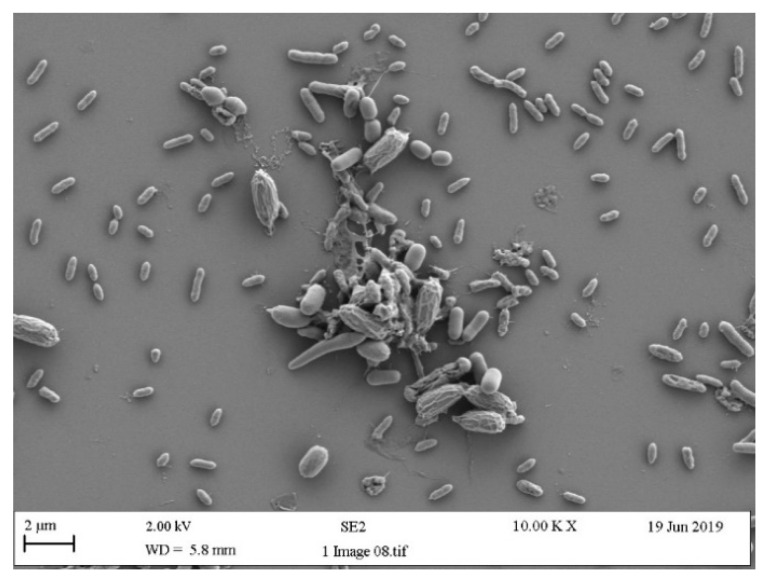
Multispecies biofilm recovered following growth of contaminated debris from washing machine, demonstrating different microorganisms present.

The process of bacterial attachment to surfaces cannot be understood without considering the properties of the substratum (solid surface), conditioning film (the layer that becomes deposited and adsorbed to a surface on exposure to an external medium), hydrodynamics (flow velocity), and the properties of the cell surface [24]. Stainless steel, polypropylene, and EDPM all possess different surface properties. Generally, when determining the effect of surface properties on bacterial retention or biofilm formation, parameters such as topography, hydrophobicity, and chemistry are measured, although other factors such as hardness may also be included [34]. It has been proposed that the topography of the surface is one of the main factors that contributes to increased bacterial contamination of a surface [29,35]. It has been demonstrated that on stainless steel, the surface chemistry affected the overall distribution of bacteria retained, whereas surface topography influenced the pattern of cell retention [29]. However, on polypropylene disks, it has been demonstrated that bacterial colonisation of microplastics was affected by both the physicochemical properties of plastics and the physiological properties of colonising bacteria [36]. In other work on four types of plastics, including polyethylene (PE), polypropylene (PP), polyethylene terephthalate (PET), and polyvinyl chloride (PVC), the results showed that the number of bacteria that adhered to PE and PVC surfaces was much greater than that attached to PP and PET, and the surface hardness of the plastics was found to be the key factor dominating the adhesion of bacteria onto plastic surfaces [37]. However, it should be noted that in different systems, such results are dependent on the individual properties of the surfaces, as in each individual environment, the cells will act in different ways since the equilibrium distance between two interacting bodies will be a result of reversible interactions [38]. Hence, if surfaces with very similar surface characteristics are compared, the findings of the effect of the surface properties on biofouling may be different from those that test surfaces with diverse surface properties. Despite this, biofilms can be recovered from many different types of surfaces in the washing machine (Figure 4).

### 3.2. The Effect of Conditioning Films

**Figure 5:**
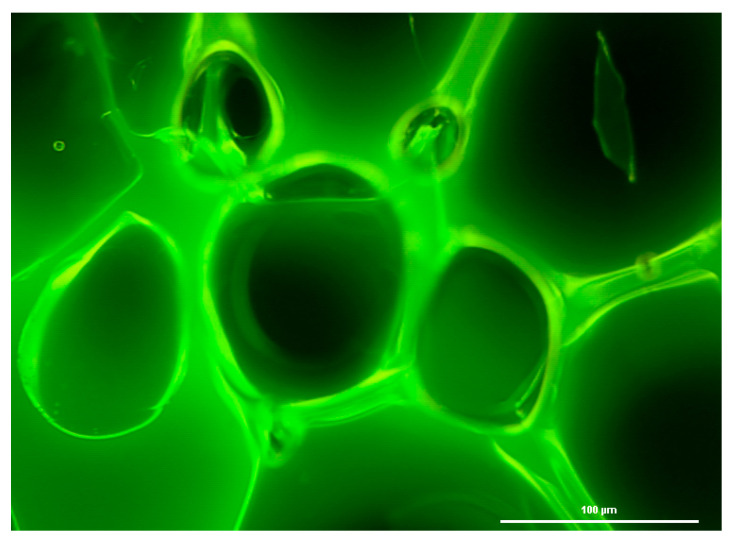
Casein stained on glass demonstrating heterogeneity of adsorbed material across the surface.

When a surface first encounters a moist environment, molecules quickly adsorb onto the surface and can change the chemical and physicochemical properties of the surface interface. The production on a surface of a conditioning film from natural waters has been shown to affect substratum properties [39], and this is an important factor to consider in washing machines. It has been demonstrated that the surface wettability can change after immersion in water for a few hours [40]. The nature of the chemical characteristics of surfaces is affected by the surrounding environment [41,42]. Research which investigated how conditioning films formed onto two hydrophobic materials used as pipe liners found that following immersion for 24 h in tap water, there were changes in the roughness and hydrophilic/hydrophobic balance of the surfaces [43]. When AISI 316L stainless steel was conditioned with natural seawater, it was found that the chemical composition of the molecules first interacting with stainless steel were proteins which adhered very rapidly, closely followed by carbohydrates [41]. On surfaces that were conditioned, biomolecules including carbohydrates, uronic acids, proteins, humic acids, and fulvic acids have been reported [44,45,46,47]. In addition to their diversity, biomolecules found to be adsorbed to surfaces were not a continuous film but a heterogeneous deposit, whose average thickness varied widely [41]. This increases the complexity of the conditioning film and subsequent surface properties because different inorganic and organic molecules adsorb to different surfaces in different patterns and clusters [48] and do not necessarily bind in a homogeneous patten across the surface (Figure 5).

The properties of the conditioning film are important since their adsorption results in chemical modification of the surface, which in turn affects microbial attachment [46,49,50]. Shallow and deep groundwaters were evaluated for their ability to generate conditioning films which affected bacterial adhesion to sandstone, shale, andesite, polypropylene, and stainless steel, and it was demonstrated that most conditioning films modified the retention of bacteria, but attachment of the organism did not correlate with the water contact angles [39]. In addition, conditioning films adsorbed from standing bore waters were often found to retain bacteria in different numbers than coatings derived from pumped bores [39]. Further, it has been suggested that even in drinking water systems with relatively low organic matter (dissolved organic carbon < 1.1 mg/L), the potential of formation of a conditioning biofilm is important [43]. When drinking water biofilm samples were formed on glass coupons in a rotating disc reactor, it was found that during the formation of a conditioning layer, surface hydrophobic forces increased, and the range of characteristic hydrophobic forces diversified with time, becoming progressively more complex in macromolecular composition, which in turn triggered irreversible cellular adhesion [40]. The chemistry of the molecules that absorb to the surface is also of importance because the biochemistry of the adsorbed layer affects the cleanability of the surface since carbohydrates are easy to remove, oils are more difficult to remove, and proteins are very difficult to remove [51].

## 4. Microorganisms Found in Washing Machines

The attachment of bacteria onto a surface is also influenced by the properties of the cell surface. It has been suggested that interactions of bacteria with one another on a surface strengthen the degree of attachment to the surface. It has also been demonstrated that the presence of flagella, pili, and fimbriae; the production of glycocalyx; the chemical structure; shape; and hydrophobicity influence and impact the extent and rate of the attachment of microbial cells to a surface [24,52]. For example, O’Toole and Kolter [53] reported how *Pseudomonas aeruginosa* pili was involved in a type of surface-associated twitching, which was required for the aggregation of cells into microcolonies.

The microbial contamination of domestic appliances has become an important subject of interest among industry and users since microorganisms have been found to inhabit surfaces of washing machines [18]. The microorganisms living inside household devices prefer the structure of a biofilm community to ensure survival and reproduction [54]. Bacterial attachment and biofilm formation inside a washing machine have been found to be abundant at places where the contact with water is almost permanent [15]. The possible consequences of microorganisms surviving in washing machines are still largely unresolved, but biofilm formation in washing machines and fabrics can be responsible for malodour [3]. Biofilm formation may become obvious to users if the amount present is too high and becomes evident as a visible biofilm or results in unpleasant odours from washing machine. Two major sources of contaminants are thought to be responsible for biofilm formation in a washing machine [55]. The human body serves as the first source, with bacteria entering the washing machine via worn clothing and household linen such as from textiles colonized with microbiota from skin and mucosa, and the second one is via the environment, which may come from influent water during the washing process [15,54].

**Figure 6:**
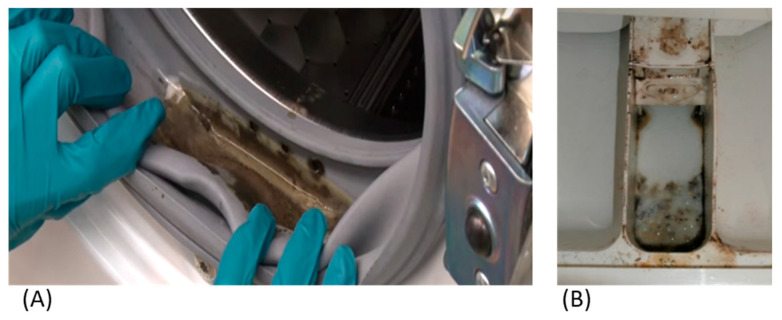
(**A**) Biofilm formation on inner rubber seal door. (**B**) Polymer washing machine detergent drawer showing biofilm formation.

The humid environment present on the inner rubber door seal, which sometimes retains water after completion of a washing cycle (Figure 6A), or the detergent drawer (Figure 6B) is provided with a constant supply of nutrients and provides excellent conditions for microorganisms in washing machines to proliferate. In a study by Bockmühl [55], it was observed that microorganisms such as viruses and faecal pathogens could be present inside a household washing machine under certain conditions. However, Proteobacteria have been found to be the predominant phylum of bacteria recovered from washing machines [54]. In addition, microorganisms isolated from washing machines have been found to include bacteria of the genera *Acinetobacter* spp., *Bacillus* spp., *Brevundimonas* spp., *Micrococcus* spp., *Staphylococcus* spp., and *Pseudomonas* spp. [56]. When biofilms were sampled from 11 washing machines from four countries and three continents, 30% were found to contain potential human pathogens, including *Pseudomonas aeruginosa* and *Klebsiella pneumoniae* [15]. *Pseudomonas putida* recovered from a washing machine was found to produce large amounts of biofilm, and it also required higher detergent concentrations than the type strain to provide effective disinfection; in addition, the recommended detergent concentration did not completely clean surfaces from cell debris and exopolymeric substances [15]. Thermophilic bacteria have also been found to be more common in certain washing machines [54,57].

There have been studies that have suggested that contaminated fabrics may result in the transmission of pathogens which could lead to infections [2,58]. Outbreaks of illness have been associated with laundering practices, particularly in healthcare facilities [1,59,60], and these include the transmission of bacteria, fungi, and viruses. Biofilms recovered from washing machines have been shown to be composed of as many as ninety-four different microorganisms, approximately a third of which could be considered potential human pathogens, including *Klebsiella pneumoniae*, *Pseudomonas aeruginosa*, *Serratia marcescens*, and *Citrobacter freundii* [15]. Work on identifying microorganisms between cotton wash cloths under standard cold water conditions in laundromat machines found that a number of opportunistic human bacterial pathogens (*Pseudomonas aeruginosa*, *Enterobacter* spp., *Enterococcus faecium*, *Klebsiella pneumoniae*, *Staphylococcus aureus*, and *Acinetobacter baumannii*) were recovered [34]. In addition, the fungal bioburden was found to be lower than the bacterial bioburden and was composed of non-pathogenic fungi, suggesting that public washing machines could be a source of non-pathogenic and pathogenic microbial contamination of laundered garments [34]. An outbreak of *Bacillus cereus* bacteraemia occurred at Jichi Medical University Hospital in 2006, and it was found that the source of this outbreak was contamination of hospital linens, and that the *B. cereus* was transmitted from the linens to patients via catheter infection [61]. In a German paediatric hospital, thirteen newborns and a child were colonized with an extended-spectrum beta-lactamase-producing *Klebsiella oxytoca*, and it was found that isolates were in the detergent drawer and on the rubber door seal of a domestic washer-extractor machine and on clothing, and this work demonstrated that the washing machine was a reservoir and that the fomite was the route of transmission for these multidrug-resistant bacteria [62]. The transmission of viruses such as hepatitis A virus, vaccinia virus (smallpox virus), and hepatitis B virus have been shown to be transmitted by textiles [59]. Fungi are of particular concern since apart from the transmission of fungal species such as *Microsporum canis* and *Tinea pedis* that result in diseases such as dermatitis and athlete’s foot [59,63,64] fungal species can be particularly life-threatening to patients that are immunocompromised [2,65].

## 5. Microorganisms on Fabrics

**Figure 7:**
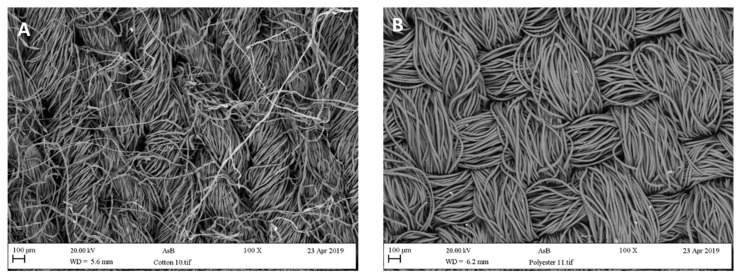
Different properties of fabrics that can become biofouled during the laundering process: (**A**) cotton and (**B**) polyester.

Clothes and textiles represent the main articles cleaned during the laundry process. A clear characteristic of the current textile market is the vast availability of different products. Three types of material can be seen to account for most sales: cotton (40%) (Figure 7A), wool (10%), and polyester mixes (50%) (Figure 7B) [66]. However, microbial contamination has been found on many laundry items and fabrics [4,19,54]. Many factors may influence the removal (detachment and/or inactivation) of microorganisms and also influence the establishment of a resident microflora in washing machines and on clothing. This is important to note as the types of bacteria in the microbial communities will influence the production of malodours [2].

Textile surfaces can enable bacteria to attach and spread along the fibres and represent the main substrate of the laundering process when they are introduced into the washing machine. It is well known that bacteria from human skin, such as microbiota from armpits or bodily excretions from underwear, can be transferred to clothes [1]. *Acinetobacter* spp. have been retrieved from textiles, and *Corynebacterium* spp., *Staphylococcus* spp., *Propionibacterium* spp., *Pseudomonas* spp., and *Micrococcus* spp. have been identified on laundered cotton samples [67,68,69]. Pathogenic bacteria causing enteric disease (*Salmonella* spp.) and skin infections (methicillin-resistant *Staphylococcus aureus*; MRSA) have also been recovered from textiles. In one study, salmonella was detected in 15% of household sponges in the United States and 3% of hand/face towels [70]. Faecal bacteria have been found to be common in undergarments of both children and adults [71]. In addition, it has been reported that *Pseudomonas aeruginosa* and *Acinetobacter* spp. could grow in clothing after laundering the clothing of wastewater treatment workers [72].

## 6. Malodour Generated in Washing Machines

Malodour generation from clothes and the washing machine itself is another issue, as bacteria produce volatile organic compounds (VOCs) that can be detected by users [19]. Personal malodour arises from the production of volatile compounds that are generated by the action of microorganisms breaking down the components of sweat, human skin cells, and secretions [73]. Sweat, sebum, and bacterial metabolites are adsorbed on the clothing through contact with the skin and can serve as precursors for malodour [56]. Studies have shown that an abundance of *Corynebacteria* spp. has been correlated to body malodour and that *Corynebacterium tuberculostearicum*, *Staphylococcus hominis*, and *Anaerococcus* spp. are also important bacteria involved in odour formation [74]. Characteristic malodour volatile compounds that can arise from these processes include ammonia, hydrogen sulphide, and short-chain fatty acids. In addition, compounds from soiled clothing that lead to malodour include butyric acid, dimethyl disulfide, dimethyl trisulfide, 2-heptanone, 2-nonanone, and 2-octanone [73]. Sulphurous compounds contribute to the axillary odour, giving it its typical onion-like and musky scent, and bacterial dipeptidases and C-S-lyases can lead to the release of mercaptoalcohols [56]. Lactic acid and glycerol released from triacylglycerides by bacteria result in acetic acid and propionic acid [75,76], and the breakdown of leucine produces an acidic malodour [56]. 4-methyl-3-hexenoic acid can be produced by *Moraxella osloensis* and is a frequently detected odour component of washed laundry that has an “off” smell [77,78].

The nature of the fabrics may also influence the perception of malodour. For example, cotton is polar and adsorbs aldehydes in high quantities, while polyester, due to its lower polarity, adsorbs less moisture [69,79,80]. However, the hydrophobic nature of polyester results in a strong adherence of fatty acids and aromatic compounds [80]. Polyester and polyester blends exhibit, in general, higher malodour intensities in comparison to cotton and wool [80,81,82,83]. Different survival times were found by Kampf [84] as bacteria at room temperature survived longer on polyester compared to fabric cotton or mixed fibres. Munk et al. [3] found that the odour formation in cotton swatches and the bacterial count of the wash liquor from cotton swatches were greater than the odour formation and bacterial count from polyester swatches. In addition, the type of material has been shown to affect the efficacy of bacterial removal, since the release of microbes is influenced by their structure, fabric type, and thickness. For example, it was more difficult to remove bacteria from bath and face towels because of their thickness [2].

Studies on the effectiveness of detergents in removing microorganisms from specific materials have shown that detergents are well known to play a role in reducing the microbial load of laundering through the release of microbes attached to fabrics and the inactivation of microbes sensitive to detergents. Cationic surfactants are considered to be excellent antimicrobial agents (although they are generally incompatible with laundry detergents), whilst anionic and non-ionic surfactants have excellent detergent properties [85]. This is of importance since the removal of bacteria from surfaces and fabrics will result in a reduction in malodour production. Cyclodextrins have been used to absorb malodours in the laundry environment [86] and to improve perfume delivery and perception. Cyclodextrins can be used in granular detergents, fabric conditioner, and in-dryer fabric enhancers, which is the most effective for perfume delivery. For malodour absorption, however, this has often been applied post-wash or pre-wash via an adjunct product, as the delivery of the active molecules is expensive and less effective in a main wash laundry detergent.

Different drying methods (tumble drying vs. air drying) can also influence microbial load. After washing, higher temperature settings and longer drying programmes when using a tumble drier can result in a lower final moisture content of the laundry, which can play an important role in inactivating microorganisms [87]. Tano et al. [88] demonstrated that without the use of tumble drying, some nonfermenting Gram-negative bacteria were found on fabrics, and it was thought that these bacteria originated from the washing machine. It has been demonstrated that air drying the laundry can effectively decrease the bacterial load, although a slow drying process results in a considerable amount of bacterial growth, giving rise to malodour formation in the laundry [3]. Odourants with branched fatty acids have been shown to dominate the odour profile after prolonged drying, and polyester swatches were demonstrated to possess a more complex odour profile than cotton due to the presence of aldehydes [3]. Miracle et al. [89] showed that copper added to a wash at levels representative of those found in water in consumer homes substantially increased the formation of known malodour molecules, but when copper chelatants were added to the wash, malodour was reduced, and when used in combination with antioxidants such as methyl3-(3,5-di-tert-butyl-4-hydroxyphenyl)propanoate, the inhibitory effect on malodour was improved.

## 7. New Concepts in Laundry: Cleanliness, Freshness, and Hygiene

Modern laundry includes the use of effective detergents and advanced technological machinery. Washing habits have changed over the last century, and washing practices in different countries of the world vary substantially. Whilst in the past, laundry practices were associated with removing dirt from clothes, this has advanced to consumers requiring products to provide improved sensory properties after the wash, such as perceived freshness, hygiene or disinfection, cleanliness, and deodorisation of the fabrics [90]. Cleanliness can be defined after laundering in terms of the time interval that clothes can be worn without negatively affecting the consumer’s health [91]. Freshness has become one of the most important aspects in laundry, as well as the evaluation of the cleanliness of clothes by assessing visible stains and odours both before and after the wash [92]. However, the perceived “cleanliness of clothes” depends on the influence of many other factors, for example, water hardness, the type of the detergent used, the level of soiling in the garments, the washing programme, and the duration of the wash [93].

The concept of freshness has also impacted and changed the lifestyle of consumers, which can drive them to adopt different ways to clean their clothes. A vast range of products have been introduced recently in the global market to enhance the quality and freshness of clothes and related items. Laundry scent boosters have been introduced into the market and can be found in various forms, including beads, liquid, powders, and others, and laundry scent crystals and beads have shown a rise in demand in recent years [94]. Such items are popular since fragrances have the ability to enhance the user’s experience and provide an impression of fabric cleanliness and freshness. As the trend gains popularity among consumers, one goal of laundry products is to obtain pleasant smelling apparel following the washing process that will bring a sense of wellbeing to the consumer and potentially influencing a person’s mood [95,96]. This is leading to a high demand for such products, and it is estimated that this commodity accounts for 63% of the market share [95]. In light of this, consumers are now actively looking for products they can introduce into their self-care routine which contain “scent”, “fragrance”, or “freshness” as one of the product’s claims. This was identified as one of the top 10 global consumer trends in 2022 [5]. For example, practically all main wash laundry detergents have some level of perfume, to either impart this perfume to the fabrics being washed, to cue freshness to the consumer on opening the bottle, or to mask unpleasant odours associated with surfactants and other chemistries present in the laundry detergents that provide cleaning efficacy. However, there have been reports of allergic skin reactions to fragrance chemicals [97,98,99,100] and it has been suggested that this may be because of fragrance residues left on clothing from washing machine products. Although some ingredients may have the potential to produce irritation and sensitization of the skin, these agents are formulated into detergent products at very low levels which are considered safe [101,102,103]. Common allergens present in laundry detergent include fragrances, isothiazolinone preservatives, isothiazolinones such as methylchloroisothiazolinone, methylisothiazolinone, and benzisothiazolinone in detergents, surfactants and propylene glycol [104]. An example of this was found when a seven-year-old girl presented with allergic contact dermatitis due to methylisothiazolinone in a laundry detergent [105]. However, it is believed that laundry detergent is the cause of allergic contact dermatitis in less than 1% of cases [104]. This may be because common allergens present in laundry detergent, such as fragrances and isothiazolinone preservatives, are likely to become reduced to clinically irrelevant levels during routine machine washing programmes [103]. Basketter et al. [106] carried out dose–response and fabric patch tests using fragrances in ethanol–diethylphthalate, and they found that twenty sensitized participants had a reaction to a fragrance-chemical-treated fabric patch, but they only displayed minor non-specific skin reactions. Therefore, the authors concluded that fragrance chemical residues present on fabric did not present a risk of the elicitation of immediate or delayed allergic skin reactions on sensitized individuals. Marrero-Alemán et al. [107] showed that when different clothes (cotton, polyester, linen, and wool) were washed in a washing machine and treated with methylchloroisothiazolinone, methylisothiazolinone, benzisothiazolinone, or octylisothiazolinone, that it was not necessary to recommend that patients sensitized to methylisothiazolinone avoid isothiazolinones in machine detergents or fabric conditioners or to double rinse clothing during washing.

Fragrance allergies have received much attention, and the European Commission [108] addressed this by enforcing legislative changes to protect sensitized users. Twenty-six key fragrance allergens were identified, and detergent products containing these chemicals above specific threshold concentrations of 10 ppm for leave on products and 100 ppm for rinse off products; in addition, these have to be labelled with the relevant nomenclature [98,100]. A skin safety program was developed by Procter & Gamble that involved a decision tree to provide a scientifically sound safety assessment of new ingredients and finished products of laundry detergents prior to their introduction to the marketplace [103].

## 8. Changes in Consumer Attitudes

**Figure 8:**
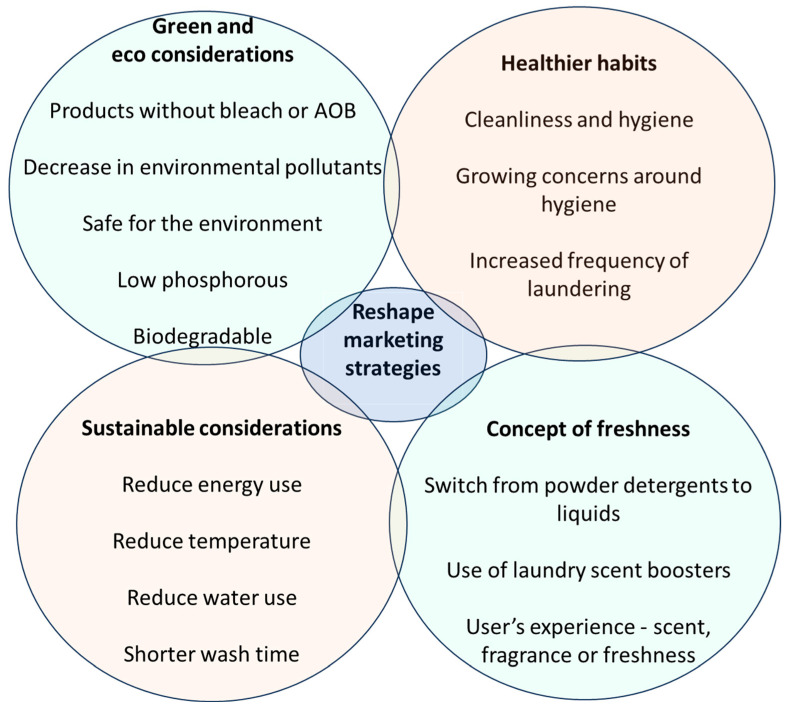
New trends in consumer habits that are driving the sustainable production of new products.

Changing consumer behaviour in relation to sustainability, environmental concerns, and maintaining a healthier lifestyle includes laundry tasks, where the need for better and eco-friendly laundry products has intensified in order to achieve sustainable laundry practices [93] (Figure 8).

Users are also becoming more conscious about healthier habits, cleanliness, and hygiene as a result of growing concerns due to rise in prevalence of infectious diseases, including the coronavirus pandemic during late 2019 and early 2020 [109]. Household cleaning plays an important role in establishing and maintaining an adequate level of hygiene [18]. Market players are using creative and innovative techniques to ensure the maximum impact of their products with claims that they will help to prevent the spread and growth of diseases. Laundry manufacturers and retailers have already begun to reshape their marketing strategies to satisfy the new and different growing demands. Moreover, not only are users and stakeholders changing their views on hygiene and cleanliness, but also several international agencies, with the help of local governments, are implementing hygiene awareness programs to prepare for future health hazards [110]. Numerous brands now use keywords which are linked to sustainable practices, such as biodegradable [111], safe for the environment, eco-friendly, and low phosphorous. This may encourage consumers to purchase products that claim to be more environmentally friendly (Table 2).

Consumer knowledge and behaviour play a crucial role in the daily basis of following sustainable laundry practices [112]. Several studies have shown that consumer behaviour is related to how people wash their laundry. Arild and Brusdal [113] conducted a detailed survey on how users in different countries in Europe such as Spain, Norway, or Greece carried out their laundry tasks, and it revealed that there were substantially different practices between the countries based on consumer attitudes and the perceived efficacy of the laundering process (Table 3).

## 9. Environmental Concerns with Laundering

In many developed countries, up to one-third of all water and energy consumption is consumed directly in the household [114], with the washing of clothes being one of the main water use activities, resulting in an average of 15% to 40% of the overall water consumption [115]. Concern has been raised regarding the chemical composition of detergents used in laundering processes, especially with respect to the discharge of contaminated effluent into water systems. Laundry has been classified as one of the routines which has a significant impact on the environment [93,116]. Many years ago, cotton was a major fabric that made up garments, and the laundry process involved high temperatures, bleach, and alkalinity. Then, consumer expectations changed and the use of phosphates was banned, and hence there was a move away from using bleaching chemicals and detergents with environmentally unfriendly chemicals [117]. Though cheap and convenient, the use of chemicals such as chlorine and oxygen bleach in detergents can have a large impact on the environment if they are not filtered out in water treatments plants, as they can end up in lakes, rivers, and groundwater. In addition, some compounds in formulations may irritate sensitive skin [118]. However, laundry detergents are becoming more environmentally friendly due to changes made in the formulations. In laundry terms, a detergent is a formulation comprising essential constituents (such as surface-active agents) and subsidiary constituents (such as perfume, colorants) [119]. The composition of a laundry detergent composition that might be used in the domestic environment is usually a formulated mixture of raw materials (surfactants, builders, boosters, enzymes, bleaching agents) that can be classified based on their properties and function [120]. However, more stringent regulations have been coming into force. For example, EU Regulation EC648/2004 [121] stipulated that all surfactants present in detergent products had to be biodegradable. The regulation was updated by Regulation (EU) No 259/2012 [122], which imposed a ban on inorganic phosphates in domestic laundry and dishwasher detergents. The aim of the regulation was to control the use of detergents and surfactants within the EU to preserve water quality and human health. Within this regulation, detergents can contain surfactants that make them clean more efficiently; however, such chemicals may damage water quality when released into the natural environment, and their use must therefore be carefully controlled. The regulation also establishes common rules to enable detergents and surfactants to be sold and used across the European Union (EU) while providing a high degree of protection to the environment and human health. The regulation allows only surfactants meeting the criterion of ultimate biodegradability to be placed on the market, either on their own (e.g., as constituent mixtures used for the manufacturing of detergents) or as ingredients in detergents. In essence, the key criteria of the regulation are that it harmonized testing methods to determine the biodegradability of all surfactants used in detergents. These cover ultimate and primary biodegradability. However, such tests must be carried out in laboratories that meet internationally recognized standards. In addition, manufacturers are responsible for ensuring their products satisfy the legislation’s requirements and must make files on test results available to the relevant authorities and an ingredient datasheet to medical staff, without delay and when requested. Information on detergents’ packaging must also be legible, visible, and indelible. This includes contact details for the manufacturer and the datasheet. Labels on detergents sold for public use must give details of recommended dosages for different washes in a standard washing machine, and national authorities may ban a specific detergent if they consider it a risk to human or animal health or to the environment. They must inform the European Commission and other EU Member States of the decision. Although there have been some suggestions for the use of alternatives in detergent design, for example, the use of Boron [123], there is little information available in this area, and this may be in part due to the commercial sensitivity.

## 10. Effect of Cycle Temperature on Hygiene

Over the last few decades, the frequency of laundering has increased, as well as the amount of clothes that people own [124]. A new trend of using lower temperatures when carrying out laundry has been observed, which has been due to the impact and awareness of how such processes affect the environment. Over the last thirty years in Britain, the quantity of washing laundry at 90 °C or above has dropped from twenty-five to seven percent [125]. Consumer behaviours changed, with laundering being carried out at a high temperature (60 °C) to a lower temperature (30 °C) [126]. In the UK, only 3% of the population was found to wash their clothes at 30 °C or below in 2002, whereas by the year 2007, this value had risen to 17% [127]. In Europe, coloured laundry is most often washed at temperatures between 30 °C to 40 °C, but in China, South Korea, Japan, and the USA, cold water is the most preferred water type [4]. However, in Norway, the cotton programme, which is often run at 60 °C, is the most used washing programme [90].

The importance of water temperature in reducing bacteria on laundered fabrics has been documented, and different studies have evaluated the antimicrobial efficacy of domestic laundering. In early studies, the investigation of the survival of microorganisms in laundered polyester-cotton sheeting by Wiksell et al. [128] using different water temperatures determined that bacteria survived washing cycles at 24 °C, 35 °C, 46 °C, and even at 57 °C. Significant differences were also found in terms of bacteriological parameters (bacteria count) when comparing cycles between low and high temperatures in commercial washing machines [129]. Honisch et al. [130] found that the trend of reducing laundry temperatures was associated with a significant decrease in hygiene effectiveness as well as high levels of cross-contamination in the water. Studies that have investigated the antimicrobial effectiveness of modern washing processes have showed that microorganisms, which mainly enter the machine through clothing or water, were reduced but not sufficiently killed during low-temperature wash cycles [4]. Such surviving microorganisms can remain inside the washing machine and either attach to different kinds of surfaces or get distributed over the wash load during the wash cycle [131]. However, although reduced inactivation of microorganisms has been reported when washes are carried out at lower temperatures, [130], work has shown that the use of lower temperatures could be compensated with a longer duration of the laundry process and the addition of disinfectant products to the wash cycle [132]. Work carried out by Laitala et al. [93] using cleaning effect tests showed that today’s detergents were suitable for low temperature washing if the correct detergent was selected, and that the result could be better at 30 °C than with a less efficient detergent at 40 °C. There have been suggested some solutions for successful agents that can reduce microbial activity when used at lower temperatures. Hydrogen peroxide and peroxyacetic acid are environmentally friendly disinfectants and have broad spectrum antimicrobial activity, low toxicity, high efficiency, and ease of use [133,134,135]. Fabrics repeatedly washed in a household washing machine using a liquid detergent with the addition of a 3% stabilized hydrogen peroxide solution were found to achieve disinfection activity but only if the solution was added in the main wash [133]. Works by others have shown that the antibacterial activity of hydrogen peroxide at 30 °C showed excellent growth reduction of bacteria but was time-dependent, with the highest antibacterial activity obtained during the main wash with a duration of 43 min [132]. One study investigated the antimicrobial effect of a solid market detergent containing activated oxygen bleach laundry detergents and liquid market detergents with regard to time and temperature in domestic washing machines and found that the washing factors affected the antimicrobial effects to varying degrees, and this was dependent on the microorganism tested [136]. At the same time, machines have maintained their washing performance, and a higher portion of machines now have an automatic load detection system [137]. With the latest smart innovations, most washing machines nowadays have an “eco” mode, which is an energy-saving feature that use less energy by washing at lower temperatures for much longer than the standard cycle and less water by increasing the drum movements. The concept of the eco-friendly programs helps people to save energy, and with the use of modern detergents, it has been shown to tackle soil removal with same results as before [138]. Echotech, Ecobubble, Coldwash, or simply Ecowash are some of the names for this washing option to allow the consumer to reduce water and energy consumption while delivering the same performance for the laundry load.

Increasing numbers of washing machines sold in Europe belong to more energy efficient labelling classes, and therefore, the average energy and water consumption per load has decreased [93]. To get UK customers washing at lower temperatures, the “Turn to 30°” campaign for Ariel delivered both volume sales uplift and changed the UK’s laundry habits—88% of those that now wash at 30 °C say they do so because of Ariel [139]. The ‘Turn to 30°’ campaign was launched in 2006 and won awards in both the Leading Edge and Ethical categories in the 2008 Marketing Society Awards [140]. The environmental awakening campaign brought about a shift in customer perceptions and behaviours—for both commercial and social benefit. However, work in South Africa using a cross-sectional survey to assess the relative importance of various environmental attributes in relation to washing machines found that the green features of the product must also perform competitively in terms of non-environmental attributes [141].

## 11. The Development of Detergents and Surface Hygiene

With the washing temperatures being reduced during the laundry processes, there is an increased interest in measuring the antimicrobial action of laundry detergents, since optimized chemistries could be an important means to compensate for lowering the temperature during wash cycles [142]. This is important since an innovative detergent design should still be able to reduce bacterial load when used at lower temperatures. It has been demonstrated that the use of premium detergent that contains bleach and temperatures at 40 °C, 60 °C, or 80 °C produced an 8-log reduction in bacterial contamination, while cycles at 30 °C that used liquid or gel detergents that did not contain bleach resulted in a lower log reduction of microorganisms [59]. Hence, to maintain the hygienic status of a washing machine and reduce the incidence of malodour, bacterial loads need to be kept low. Bleaching agents are considered one of the major groups of ingredients that may determine the antimicrobial efficacy of detergents [55]. The most commonly used activators are tetraacetylethylenediamine (TAED) and sodium nonanoyloxybenzenesulphonate (SNOBS), with the former used across Europe and the latter in the United States and Japan. Although both activators are widely used in laundry detergents to enable bleaching at low temperatures, TAED has been suggested to be the only industrialized activator that presents the benefits of being non-toxic, non-sensitizing, and biodegradable. However, a major disadvantage has been observed from these bleach activators, as they go under hydrolysis under basic conditions [143], which is why they are generally used in powder formulations and not in liquid detergent formulations.

In addition to the technical aspects of the laundry process, consumer behaviour has the potential to influence the laundry process in terms of hygiene and antimicrobial efficacy. Several studies dealing with domestic laundering processes [55,113,130,136] have shown the preventive effect of activated oxygen bleach detergents at temperatures lower than 30 °C. Yet, there has been an increase in consumer use of liquid detergents and pods rather than the use of more traditional washing powders. However, liquid detergents do not contain bleaching agents, and so, potential negative effects such as microbial contamination or malodour in washing machines and freshly washed clothes have been reported [3,18,144]. The switch from powder detergents to liquids has also shown a significantly decrease in the antimicrobial effect [1,130]. A significant transfer of microbial cells has been reported with bleach-free detergents at temperatures lower than 30 °C and even at 40 °C when using shorter cycle durations [55,113,130,136]. Detergent manufacturers have responded to these changes by increasing the use of non-ionic surfactants in their washing formulations because they bind to proteins and are noted for their effectiveness at low temperatures, as well as by adding enzymes and bleach activators [145].

Recent biotechnological-based innovations include biobased laundry detergents and non-bio laundry detergents, which are those with or without enzymes, respectively (Table 4). Microbial enzymes are widely used as additives in laundry detergents [120] as part of what is known as biological cleaning or biotechnology. Detergent compositions with enzymes can function at lower temperatures, thereby reducing energy consumption [146]. Additional improvements can be reached by continuing the replacement of harmful chemicals with biobased, readily degradable ingredients [147]. 

Many of today’s consumers are turning to biodegradable laundry detergent brands as part of the new influence to be more “green” or “eco-friendly”. However, early sustainable and biodegradable products suffered from a misconception that they could not be as effective as standard alternatives [148]. These new biodegradable detergents are designed to be gentle on fabrics, preserving textile’s colour and texture while effectively removing dirt and tough stains. The use of micro-nano bubbles in the washing process has been suggested as an eco-friendly alternative, effectively reducing washing time, mechanical force, and detergent consumption compared to conventional methods [149].

**Table 4:** Enzymes used in laundry detergent formulations and their target and function [150,151,152,153].

Enzyme	Stain Origin	Laundry Target	Function
Proteases	Protein-based	Grass, blood, egg, human sweat	Clean appearance, fabric whiteness
Amylases	Starch-based	Pasta, potatoes, baby food, rice, corn	Improve cleaning efficiency, prevent particulate deposition on starchy soils
Lipases	Fat-based	Butter oil, human sebum, salad oil, sauce	Remove fatty residues
Cellulose	Soils	Soot, clay, rust	Fabric and colour care; colour brightening, softening, and soil removal
Mannanase	Mannans	BBQ sauce, chocolate, ice cream, toothpaste, deodorant	Stain removal and preventing fabric damage
Pectate lyase	Pectin-based	Fruits, vegetables, jams, food containing thickeners	Cleaning performance and stain removal; prevent stains set into the fabric

## 12. Conclusions

Devices such as washing machines have been proven to be an ideal habitat for microbial communities to thrive. Laundering practices and consumers’ demands have changed, influenced by social, cultural, environmental, and economic norms. The difference in the surface properties and incoming contaminants and microbes from water and fabrics to be laundered provide a rich milieu that can support biofilm growth and provide a challenge for manufacturers of laundering detergents to manage. The latest trends towards using lower temperatures to reduce the environmental impact and increase cost savings, in addition to the switch to the use of liquid detergents instead of powders, have led to suggestions that there might be an increased risk of microorganisms surviving the laundering process. This has led to concerns about bacterial contamination, especially since biofilm formation can be seen and detected by the naked eye by the consumer and can produce malodour in the washing machine or clothes even after a wash cycle. Practical interventions for minimizing biofilm formation include using detergents at the correct temperatures. Although there are suggestions for different biotechnological approaches that have the potential to reduce biofilm formation in washing machines and on clothing, very few have been tested in washing machines in the presence of soil from clothes and with different detergents and machine settings. However, work on producing more ecofriendly machines, and detergents whose components are less harmful to the environment, are being developed. To meet the changes in consumer needs, an understanding of the interactions of microorganisms with surface components and detergents is needed to enhance the efficacy of new antimicrobial cleaning regimes.

## Figures and Tables

**Table 1:** The different stages of biofilm formation.

Stage	Description	Visual Image
Attachment	Bacteria are reversibly attached to the surface	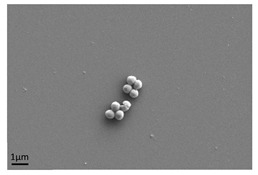
Adhesion	Bacteria begin to adhere more firmly to the surface	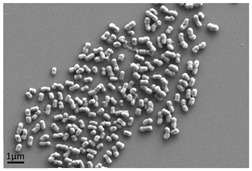
Retention	Bacteria become retained on the surface and cannot be removed under flow	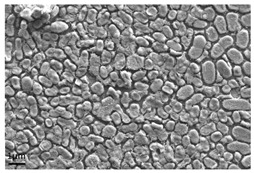
Initial biofilm formation	Bacteria begin to proliferate and produce EPS	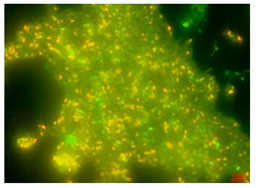
Mature biofilm formation	Biofilm becomes established and can be visibly seen on components	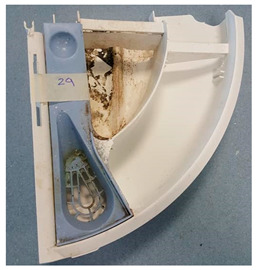

**Table 2:** Keywords use by commercial brands linked to sustainable practices.

Terminology	Definition
Carbon footprint	The amount of carbon dioxide released into the atmosphere as a result of the activities of a particular individual, organization, or community
Carbon neutral	No net release of carbon dioxide into the atmosphere
Net zero emissions	When emission of greenhouse gases and removal are in balance
Biodegradable	A substance or object capable of being decomposed by bacteria or other living organisms and thereby avoiding pollution
Sustainable	Meets the needs of the present without compromising the ability of future generations to meet their own need
Eco-friendly	Living in a way that is not harmful to the environment
Chemical free	Not involving the use of artificial chemicals, especially pesticides or fertilizers
Natural	Existing in or derived from nature
Toxic	Degree to which a chemical substance or a particular mixture of substances can damage an organism
Sustainable standards	Voluntary guidelines used by producers, manufacturers, traders, retailers, and service providers to demonstrate their commitment to good environmental, social, ethical, and food safety practices
Sustainability	Avoidance of the depletion of natural resources in order to maintain an ecological balance
Environmental management	Positive and negative impacts to the physical environment
Green	Less harmful to the environment
Renewable energy	Energy from a source that is not depleted when used

**Table 3:** Consumer requirements towards laundry tasks.

Country	Germany	Finland	Netherlands	Norway	Spain	UK	Italy
Detergent	Powder heavy duty detergent with or without bleach	Unscented detergent	Compact powder	Compact powder	Traditional powder	Concentrated liquid/gel	No data
Use of fabric conditioner	No	No	No data	Yes	Yes	Yes	No data
Temperature	40–45 °C Average: 44.5 °C	40–60 °C Average: 45.1 °C	40 °C Average: 49 °C	45–50 °C Average: 48.4 °C	15–30 °C or 30–40 °C	30–40 °C	40.4 °C
Frequency of washes per week	4	3.7	4.8	6.5–8	3	4	3.7
Energy use per household/year (KWh)	89.7	106.1	96.6	135.2	66.4	115.6	112.7
Programme (more common used)	Cotton	No Data	Laundry is selected according to colour; woolen and silk garments are washed by hand	Cotton/ short programme	An additional rinse is observed for the most common programme	Cotton 40 °C	Cotton 40 °C

## Data Availability

Not applicable.
