# Correction: Osta-Ustarroz et al. Microbial Colonization, Biofilm Formation, and Malodour of Washing Machine Surfaces and Fabrics and the Evolution of Detergents in Response to Consumer Demands and Environmental Concerns. *Antibiotics* 2024, *13*, 1227

**DOI:** 10.3390/antibiotics14030292

**Published:** 2025-03-11

**Authors:** Patricia Osta-Ustarroz, Allister J. Theobald, Kathryn A. Whitehead

**Affiliations:** 1 Lubrizol Life Science, Vanguard House, Keckwick Lane, Daresbury, Cheshire WA4 4AB, UK; patricia.osta-ustarroz@lubrizol.com; 2 Microbiology at Interfaces, Manchester Metropolitan University, Chester Street, Manchester M1 5GD, UK

## Figure Legend

In the original publication [1], there was a mistake in the legend for Figure 7—Different properties of fabrics that can become biofouled during the laundering process: (**A**) polyester and (**B**) cotton. The images of fabrics did not correlate with the explanation in the legend. The correct legend appears below.

**Figure 7.** Different properties of fabrics that can become biofouled during the laundering process: (**A**) cotton and (**B**) polyester.

The authors state that the scientific conclusions are unaffected. This correction was approved by the Academic Editor. The original publication has also been updated.
